# Subcortical gray matter atrophy and iron deposition in patients with vascular dementia: a multimodal MRI study

**DOI:** 10.3389/fnins.2026.1843628

**Published:** 2026-07-21

**Authors:** Jun Yang, Cuiping Bao, Xuehuan Liu, Jinxia Yan, Yiming Li

**Affiliations:** Department of Radiology, Tianjin Union Medical Center, The First Affiliated Hospital of Nankai University, Tianjin, China

**Keywords:** brain MRI, brain volume, iron deposition, subcortical gray matter, vascular dementia

## Abstract

**Purpose:**

Vascular dementia (VAD) is the second most common type of dementia worldwide. Therefore, early detection and diagnosis, along with a clear understanding of its pathogenesis are critical for mitigating disease progression. In the present study, we aimed to elucidate the associations of brain volume and iron deposition with VAD based on structural brain and iron content analyses.

**Methods:**

Fifty-three patients with VAD and 43 control participants were recruited for this study. All participants underwent the Mini-Mental State Examination (MMSE) and brain MRI scans. This study primarily focused on the volume of specific brain regions (assessed using FreeSurfer) and iron deposition (evaluated using quantitative susceptibility mapping [QSM]). Linear regression analysis was also performed.

**Results:**

Patients with VAD exhibited significant reductions in brain volume in the left putamen (β = −0.342, 95% CI: −0.581 to −0.104), left pallidum (β = −0.099, 95% CI: −0.187 to −0.001), left hippocampus (β = −0.138, 95% CI: −0.271 to −0.004), and right hippocampus (β = −0.235, 95% CI: −0.420 to −0.051). Additionally, significant increases in iron levels were identified in the left (β = 0.006, 95% CI: 0.002 to 0.010) and right (β = 0.005, 95% CI: 0.001 to 0.009) hippocampus.

**Conclusions:**

These findings indicate that brain volume reduction and increased iron levels in specific regions may be associated with cognitive deficits in patients with VAD.

## Introduction

Vascular dementia (VAD), the second most common form of dementia globally after Alzheimer's disease (AD) (Akhter et al., 2021), is clinically characterized by stepwise progression. Globally, over 50 million individuals are affected by dementia, and VAD accounts for 33% of all cases (Smith, 2017). Cognitive deterioration in VAD, which generally occurs concomitant with mood disturbances, such as depression and anxiety, has a profound impact on the quality of life and imposes a substantial burden on societal healthcare resources. VAD is a major public health challenge (Wolters and Ikram, 2019). Early detection and diagnosis of, along with achieving a clear understanding of its pathogenesis are critical for mitigating disease progression.

Recent advancements in neuroimaging techniques, have emerged as an indispensable tools for the comprehensive evaluation of individuals presenting with VAD. Magnetic resonance imaging (MRI) is a non-invasive, region-specific method used to quantitatively analyze volume changes in different brain regions which has been employed to analyze structural abnormalities in VAD (Frantellizzi et al., 2021). Brain volume in key brain regions have also been observed in neurodegenerative diseases. According to one prior study, hypothalamic atrophy is evident in early stages AD. Results from a population-based study have demonstrated that hippocampal atrophy is the significant radiological feature in patients with VAD (Scher et al., 2011). Previous pathological studies have shown that the volume of hippocampal neurons in the cornu ammonis (CA) 1, CA2 and CA4 regions were reduced in patients with VAD (Gemmell et al., 2014; Hase et al., 2024).

Iron is essential for numerous physiological processes in the brain including neurotransmitter synthesis, mitochondrial function, and myelin sheath formation. Recently, iron concentrations in the deep gray matter of the brain have garnered considerable attention in research on neurodegenerative disease-related cognitive impairment (Galy et al., 2024; Santiago Gonzalez et al., 2019). The assessment of iron deposition based on MRI has gained widespread application owing to its non-invasive nature. Although conventional T2^*^-weighted or susceptibility weighted imaging (SWI) has certain applications in the assessment of iron deposition, it is difficult to conduct an accurate quantitative evaluation of iron deposition. Quantitative Susceptibility Mapping (QSM) is a sophisticated methodological advancement that, enables the derivation of quantitative data pertaining to the magnetic susceptibility of brain tissue via the reconstruction of a susceptibility map (Sun et al., 2018; Wang et al., 2025). This method has been widely applied to assess iron deposition in various diseases. Iron deposition due to QSM is correlated with cerebral vascular degeneration and cognitive decline (Jia et al., 2023; Kim et al., 2022). A recent study reported that iron deposition may be related to cognitive dysfunction in subcortical vascular mild cognitive impairment (Liu et al., 2025); however whether iron deposition is genuinely associated with vascular dementia remains to be investigated.

In the present study, we aimed to clarify the associations of brain volume and iron deposition with VAD based on structural brain and iron content analyses, we sought to: 1) evaluate the brain volume in VAD using T1-3D-MPRAGE, and 2) analyze the relationship between iron deposition and VAD using QSM. This study highlights the importance of QSM as a pathologically validated method for the evaluation of brain iron deposition, offering a reliable and innovative imaging tool for the assessment of this condition.

## Materials and methods

### Participants

Participants were recruited from Tianjin Union Medical Center between August 2019 and July 2024. This study was approved by Tianjin Union Medical Center Medical Research Ethics Committee (B156) and written informed consent to participate was obtained from all participants.

This study enrolled patients with VAD who underwent brain MRI and met the following criteria: (1) met the diagnostic criteria for VAD according to the DSM-5 criteria for Major Vascular Neurocognitive Disorder (operational definitions provided in Supplementary Table S1) (First, 2013), (2) age ≥50 years, (3) with qualified brain MRI, (4) the region of research interest had no infarction lesions, and (4) with complete clinical information. Patients with brain trauma or tumors were excluded.

### Clinical data collection

Clinical data were collected by searching the Hospital Information System (HIS), Picture Archiving and Communication System (PACS), and participant self-reports. Patient information included age, sex, height and weight. Body mass index (BMI) was calculated as weight (kg) divided by height squared (m^2^). Brain trauma and brain or other site tumors were evaluated according to the medical records. Global cognitive function was assessed by using the Mini-Mental State Examination (MMSE).

### MRI data acquisition

Whole-brain anatomical images were acquired using a 3T MRI scanner (Skyra, Siemens, Healthcare, Germany) equipped with a 20-channel head-neck coil. Each participant underwent a comprehensive neuroimaging protocol, that included T1-weighted imaging (T1WI), T2-weighted imaging (T2WI), diffusion-weighted imaging (DWI), T2-weighted fluid-attenuated inversion recovery (T2-FLAIR), 3D T1-weighted magnetization-prepared rapid acquisition with gradient echo (MPRAGE), and QSM. Following acquisition, all images were meticulously evaluated by an experienced neuroradiologist to ensure the absence of any motion-related artifacts and severe image degradation on the routine sequences, particularly T1-MPRAGE and QSM. The respective sequence parameters of these modalities are presented in Supplementary Table S2. Volumetric and iron deposition analyses were restricted to the caudate, putamen, pallidum, and hippocampus, both of which were restricted to an *a priori* selected region of interest (ROI). These structures were selected with a clear pathophysiological rationale based on prior evidence that consistently implicated them in vascular cognitive impairment. Because QSM analysis, which is limited by the feasibility of manual ROI delineation, was completed only for these ROIs, volumetric analysis was performed for the same ROIs to maintain consistency between the two modalities.

### Data of brain volume processing

First, all data were converted to NIfTI format using Mricron software; then the images were processed using FreeSurfer version 7.0.0 (https://surfer.nmr.mgh.harvard.edu/) with default settings. Processing involved the following steps; motion correction, intensity normalization, skull stripping, transformation to the MNI template, segmentation, creation of the cortical surface and parcellation. Brain volumes, assessed using the DKT-atlas template, included the caudate, putamen, pallidum, hippocampus, and total brain.

### QSM reconstruction

**Figure 1:**
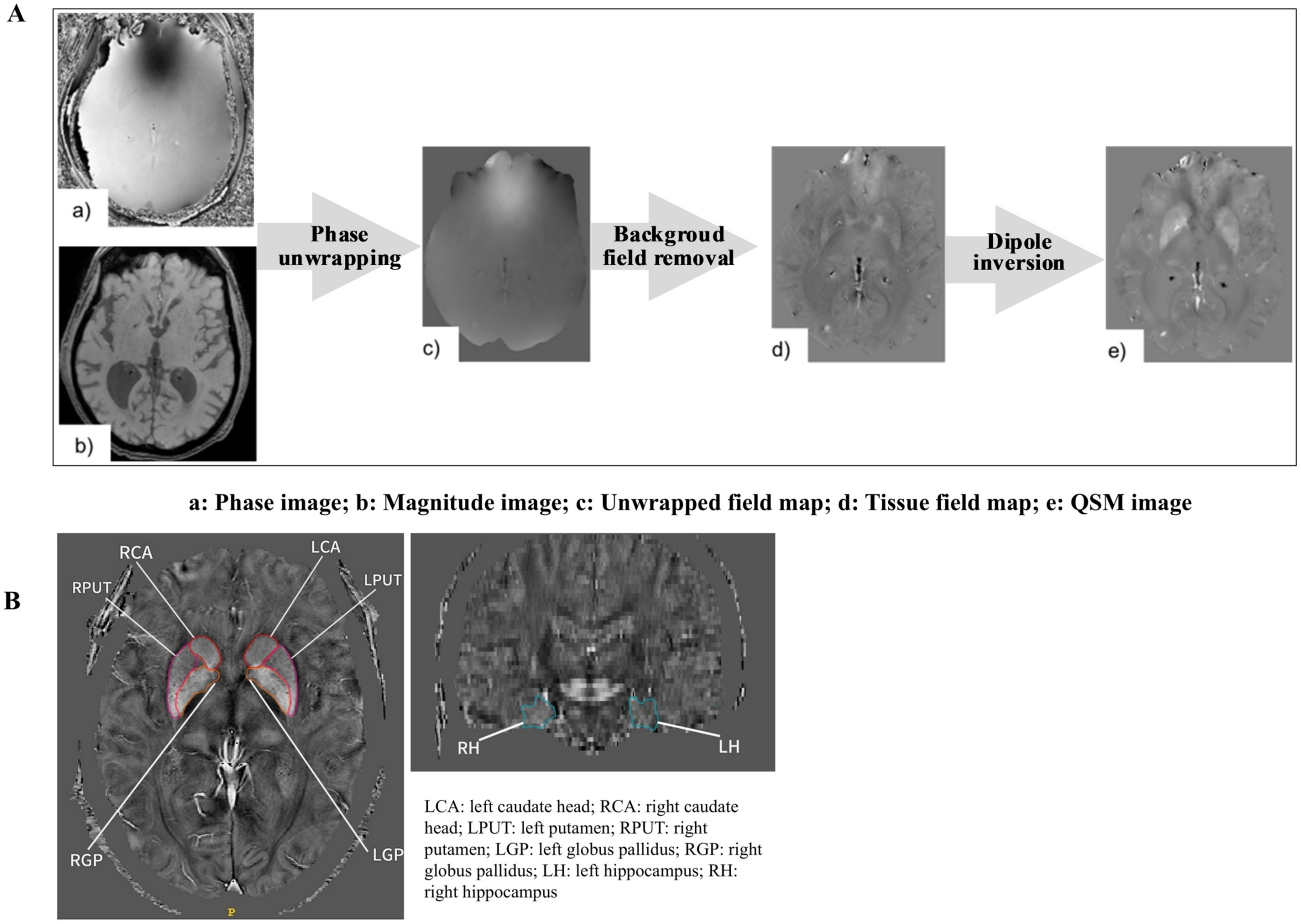
QSM image analysis workflow and schematic diagram of ROI(s). **(A)** QSM image analysis workflow; **(B)** schematic diagram of ROI(s).

After acquiring the magnitude and phase images through scanning, STISuite 3.0, a post-processing software package (Duke University), was applied to generate the QSM images. The processing procedure (Schweser et al., 2017) is presented in Figure 1A. In brief, analysis was conducted as follows: Firstly, susceptibility map reconstruction is composed of three steps: phase unwrapping, background field removal, and dipole inversion. Following importation of the amplitude and phase diagrams, the susceptibility map was unwrapped using a technique based on the Laplacian operator (Bagher-Ebadian et al., 2008). Subsequently, elimination of background fields originating from sources situated outside the region of interest (ROI), which can significantly distort the calculated susceptibility maps, was conducted. Thus, the mask and ROI were separated from the background, retaining the tissue field generated by the distribution of the regional magnetic susceptibility. Using the method of projection onto dipole fields, the overall field measured within the ROI was projected onto the subspace, which was spanned by all background unit dipole fields. In the inversion step, deconvolution was conducted using the dipole kernel. This process enabled extraction of the local tissue susceptibility within the ROI from the background-corrected tissue field. Dipole inversion was then conducted on the tissue field map to reconstruct quantitative susceptibility images with excellent contrast and high tissue resolution to better delineate the ROI in the ITK-SNAP software. After the image was converted into the DICOM format from the QSM format, using dcm2niigui in the MRICron software, it was transformed into NII format from DICOM format.

## ROI selection

Previous studies have demonstrated that cerebral iron deposition negatively affects cognitive function and is associated with the stability of intelligence (Penke et al., 2012). Therefore, we selected brain regions with greater iron deposition as the ROIs to verify the relationship between iron deposition and Vascular Dementia using the QSM format. Eight brain regions were selected as ROIs: the left caudate head, right caudate head, left putamen, right putamen, left globus pallidus, right globus pallidus, left hippocampus, and right hippocampus. The anatomical distributions of these structures are presented in Figure 1B. All ROIs were chosen using the ITK-SNAP software (version 3.8, http://www.itk-snap.org) to determine their suitable positions under the guidance of experienced neuroradiologists, which revealed the largest region shown by the brain structure to mark. To ensure measurement accuracy, two adjacent slices above and below the largest slice were consecutively marked to form a three-slice measurement protocol.

## Computational susceptibility quantification

The mean magnetic susceptibility values were derived by voxel-wise averaging across the tri-slice ROI volume using QSM reconstruction algorithms. When complete, the magnetic susceptibility value for each ROI was calculated as the mean value across three consecutive slices. During the measurement process, regions with susceptibility artifacts or partial volume effects were systematically excluded. Following completion of ROI measurement, comprehensive magnetic susceptibility data were compiled for subsequent statistical analyses.

### Statistical analysis

The chi-square test was applied to compare baseline proportions, as described by the frequency and proportion. Student's *t*-test were used to compare the means of normally distributed continuous variables, described as the mean and standard deviation (SD). The Mann-Whitney U test was used to assess non-normally distributed continuous variables, with data described as the median and interquartile range (IQR). We constructed separate linear regression models for each regional brain volume measure (caudate, putamen, pallidum, and hippocampus) and each regional iron deposition measure (QSM values in the same regions), using the regional measure as the dependent variable and VAD status (dichotomous: VAD vs. control) as the primary independent variable. All models were adjusted for age, sex, and BMI as covariates. For volumetric analyses, each regional brain volume was expressed as a percentage of the total brain volume (‰) to standardize for any individual differences in head size. The results were reported as unstandardized β coefficients with 95% confidence intervals.

Sensitivity analyses were conducted by repeating the main analyses, stratified by sex and age. All analyses were performed using R software (version 3.3.1). The level of statistical significance for the two-tailed test for each hypothesis was set at *P* < 0.05.

## Results

### Baseline characteristics

A total of 53 patients with VAD and 43 controls were recruited for this study. Of these, the mean age was higher in the VAD (65.79 years) than the healthy control (59.98 years) group. Compared with control participants, patients with VAD were more likely to be male (*P* < 0.001) while no significant differences were observed in BMI. Regarding the cognitive test, patients with VAD had lower MMSE scores than control participants (*t* =14.672, *P* < 0.001) (Table 1).

**Table 1:** Basic characteristics of the participants.

Variables	Healthy controls (*n* = 43)	VAD (*n* = 53)	Statistics	*P*
Age, mean (SD)	59.98 (6.87)	65.79 (5.38)	−4.653	<0.001
Sex, *n* (%)			27.612	<0.001
Male	15 (34.88)	46 (86.79)		
Female	28 (65.12)	7 (13.21)		
BMI, mean (SD)	24.21 (2.67)	23.65 (3.08)	0.941	0.349
MMSE, mean (SD)	28.42 (1.31)	20.28 (3.76)	14.672	<0.001
Location of ischemic focus				
Left hemisphere	–	11 (20.75)	–	–
Right hemisphere	–	6 (11.32)	–	–
Bilateral hemisphere	–	36 (67.92)	–	–

### Relationship between VAD and domain-specific brain volume

**Figure 2:**
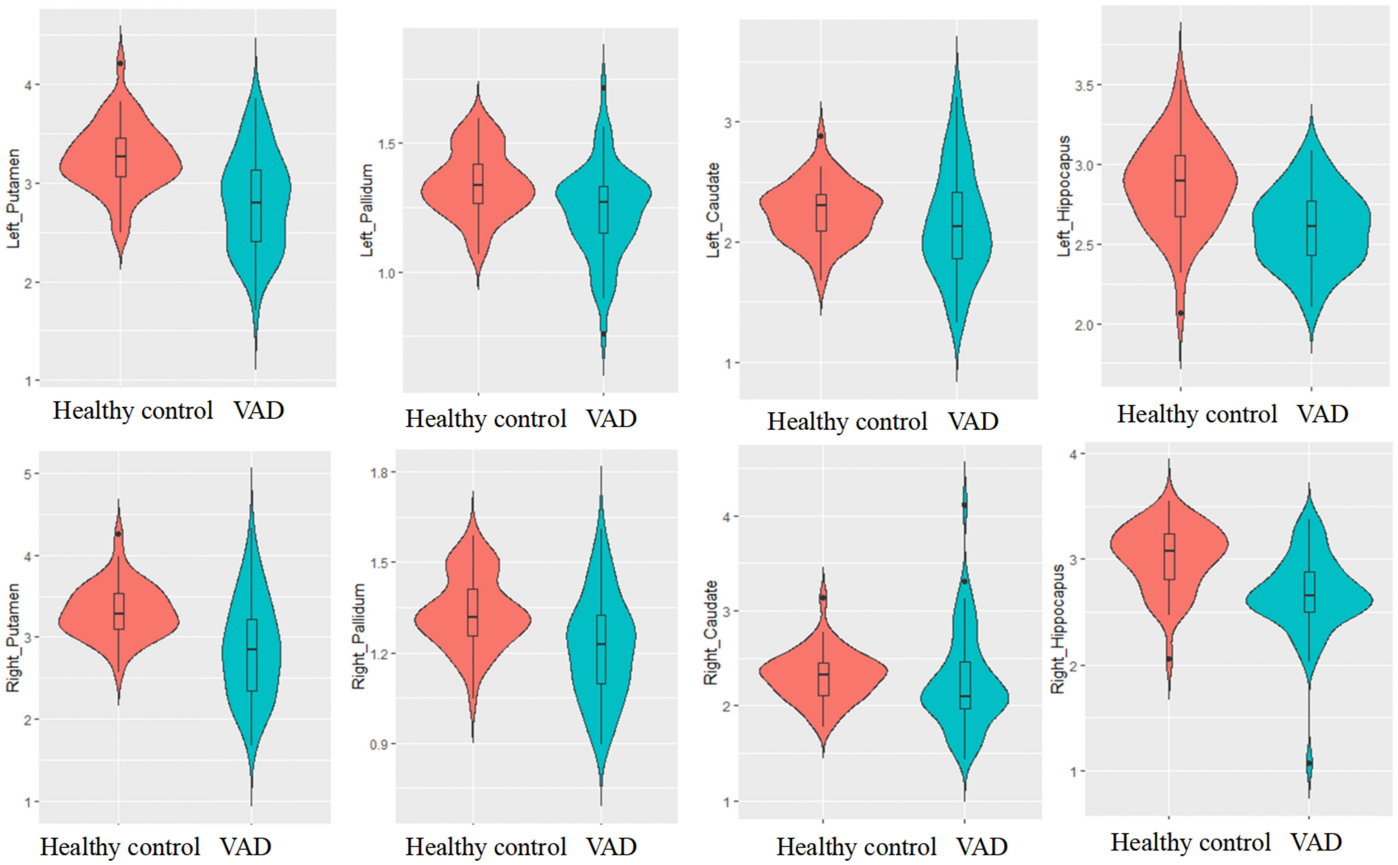
The domain-specific brain volumes between VAD and healthy controls.

**Figure 3:**
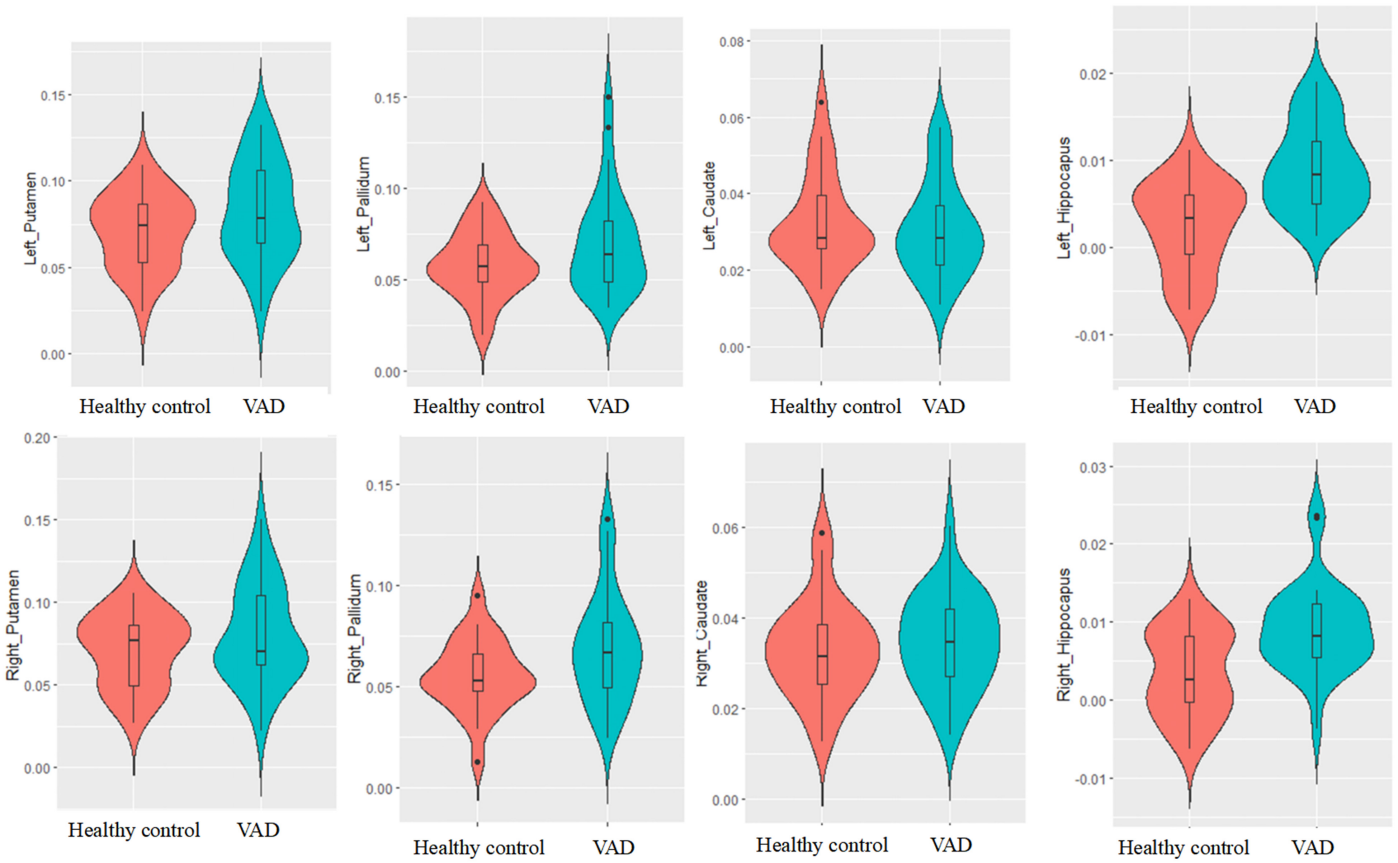
Visualization of regional brain volumes between VAD and healthy controls.

In univariate analysis, patients with VAD exhibited reduced volumes in multiple brain regions compared with healthy controls, including the left putamen, left pallidum, left hippocampus, right putamen, right pallidum and right hippocampus. However, no significant differences were observed in the volumes of the left and right caudates (Table 2, Supplementary Table S3 and Figure 2). After adjusting for age, sex and BMI, in multi-adjusted linear regression models, the association remained in the following brain regions: the left putamen (β = −0.342, 95%CI: −0.581, −0.104), left pallidum (β = −0.099, 95%CI: −0.187, −0.001), left hippocampus (β = −0.138, 95%CI: 0.271, −0.004), and right hippocampus (β = −0.235, 95%CI: −0.420, −0.051) (Table 2 and Figure 3).

**Table 2:** Relationship between VAD and domain-specific brain volume/ iron levels by multiple linear regression.

Brain regions	*n*	Basic-adjusted β (95% CI)^a^	Multi-adjusted β (95% CI)^b^
Domain-specific brain volume			
Left caudate	96	−0.096 (−0.248, 0.056)	−0.044 (−0.245, 0.157)
Left putamen	96	−0.445 (−0.622, −0.268)	−0.342 (−0.581, −0.104)
Left pallidum	96	−0.107 (−0.172, −0.042)	−0.099 (−0.187, −0.001)
Left hippocampus	96	−0.269 (−0.375, −0.163)	−0.138 (−0.271, −0.004)
Right caudate	96	−0.066 (−0.236, 0.103)	−0.056 (−0.286, 0.174)
Right putamen	96	−0.461 (−0.665, −0.257)	−0.261 (−0.532, 0.011)
Right pallidum	96	−0.119 (−0.180, −0.058)	−0.045 (−0.125, 0.034)
Right hippocampus	96	−0.351 (−0.494, −0.208)	−0.235 (−0.420, −0.051)
Domain-specific brain iron levels			
Left caudate	54	−0.002 (−0.009, 0.005)	0.001 (−0.008, 0.010)
Left putamen	54	0.011 (−0.003, 0.025)	0.004 (−0.015, 0.022)
Left pallidum	54	0.012 (−0.001, 0.026)	0.005 (−0.012, 0.023)
Left hippocampus	54	0.007 (0.004, 0.009)	0.006 (0.002, 0.010)
Right caudate	54	0.002 (−0.004, 0.007)	0.005 (−0.002, 0.013)
Right putamen	54	0.009 (−0.005, 0.024)	−0.003 (−0.022, 0.016)
Right pallidum	54	0.013 (0.001, 0.026)	0.005 (−0.013, 0.022)
Right hippocampus	54	0.006 (0.003, 0.009)	0.005 (0.001, 0.009)

^a^Basic adjustment model, adjusted for age;

^b^multivariate adjustment model, adjusted for age, sex and BMI.

### Relationship between VAD and domain-specific brain iron levels

**Figure 4:**
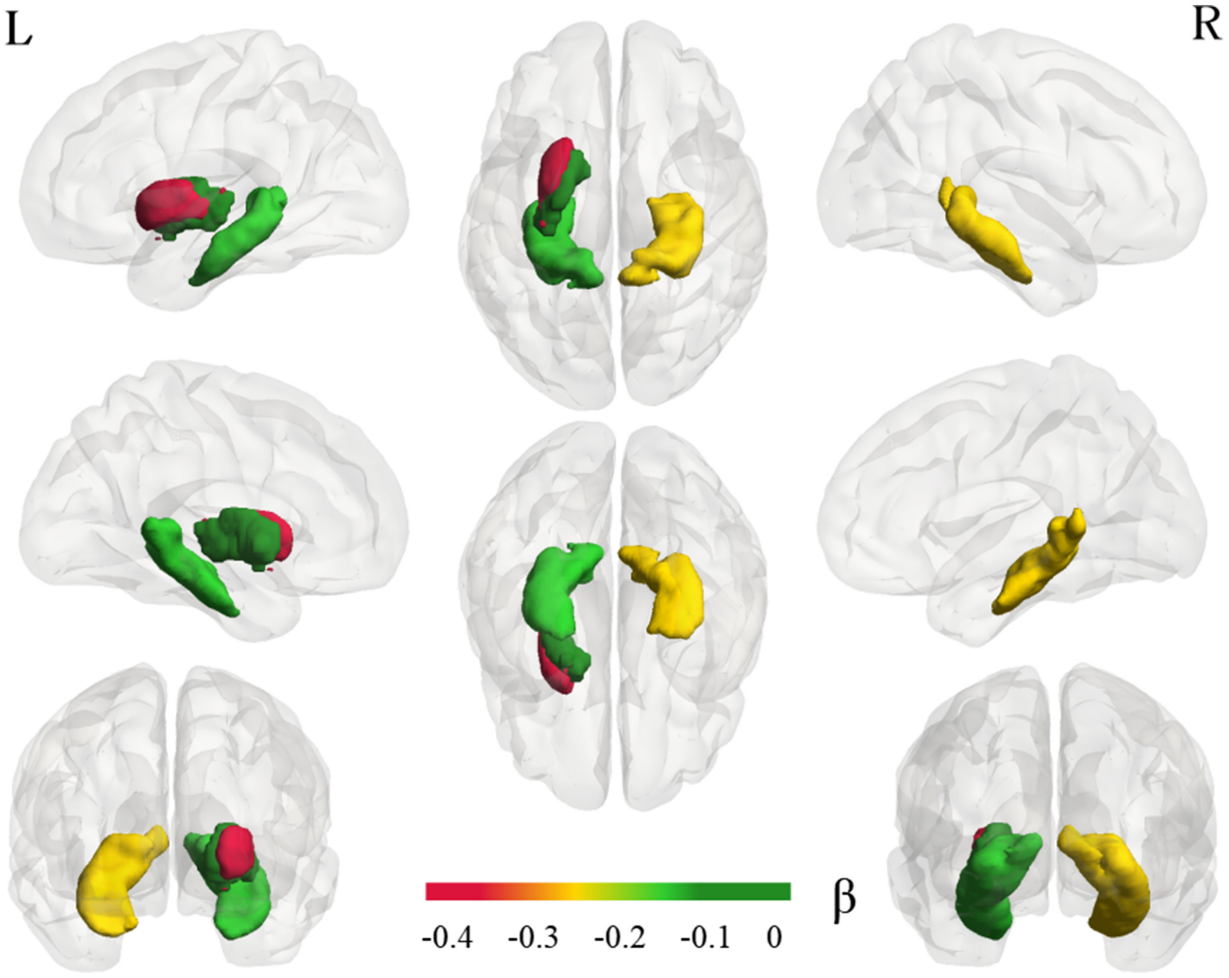
The domain-specific iron levels between VAD and healthy controls.

In univariate analysis, patients with VAD exhibited increased iron levels in multiple brain regions compared with healthy controls, including the left hippocampus, right pallidum and right hippocampus. However, no significant differences were found for the volumes of the left caudate, left putamen, left pallidum, right putamen or right caudate (Table 2, Table S4 and Figure 4). After adjusting for age, sex, and BMI, in multi-adjusted linear regression models, the association remained in the following brain regions: the left hippocampus (β = 0.006, 95%CI: 0.002, 0.010) and right hippocampus (β = 0.005, 95%CI: 0.001, 0.009) (Table 2).

## Discussion

In the present study, we used brain volume and iron levels as measures of brain atrophy and iron deposition in patients with VAD. Overall, we found that patients with VAD exhibited the following features: (1) volume reductions in the left putamen, left pallidum, left hippocampus, and right hippocampus (2) Iron deposition in the left and right hippocampi.

The putamen and globus pallidus, the core parts of the basal ganglia, play crucial roles in movement regulation and cognitive functions (Hanakawa, 2021). In the present study, atrophy of the left putamen and pallidum in patients with VAD presenting with left-sided brain ischemia was likely attributable to ischemia within the branches of the middle cerebral artery, thus affecting the neuronal and neural fibrous components within these regions. Such atrophy may be implicated in the pathological mechanisms underlying motor dysfunction and cognitive decline in patients with VAD with manifestations such as deficits in executive functioning and attention (Jellinger, 2023). Notably, although a significant volume loss was observed in the left putamen and pallidum, we did not detect any corresponding increases in magnetic susceptibility in these regions. This dissociation between structural atrophy and iron deposition indicates that the pathological mechanisms in the basal ganglia differ from those in other vulnerable regions. Indeed, the putamen and pallidum are primarily supplied by the deep perforating branches of the middle cerebral artery; as such, chronic ischemia in this territory can lead to neuronal loss, axonal degeneration, and reactive gliosis, resulting in volume reduction without necessarily involving iron-mediated oxidative stress (Donzelli et al., 1998; Fotiadis et al., 2021). Moreover, the basal ganglia physiologically contain high levels of iron, and their iron homeostatic mechanisms may be more robust or tightly regulated (Du et al., 2021; Persson et al., 2020), potentially rendering them less sensitive to pathological iron accumulation throughout the early stages of vascular cognitive impairment.

The hippocampus plays a crucial role in cognitive processes such as memory formation and spatial navigation (Planche et al., 2021). The presence of atrophy in both the left and right hippocampi of patients with VAD is consistent with prior research demonstrating that the hippocampus is particularly susceptible to damage in VAD and mild cognitive impairment (Li et al., 2016). Alterations in magnetic susceptibility are correlated with changes in iron metabolism. In neurodegenerative conditions such as VAD, elevated magnetic susceptibility within the brain tissue primarily reflects excessive iron deposition (Yan et al., 2024). In our study, we further observed that patients with VAD had iron deposition in the left and right hippocampi, which is consistent with previous findings (Sun et al., 2017). However, as hippocampal atrophy and iron accumulation have also been reported in Alzheimer's disease, we acknowledge that the observed hippocampal changes in patients with VaD may not be exclusively vascular in origin. While we excluded participants with clinical features suggestive of AD at enrollment, the absence of CSF or PET biomarkers precluded the definitive attribution of hippocampal findings.

Patients with VAD commonly present with vascular risk factors which can induce structural damage in the cerebrovascular system and precipitate endothelial dysfunction. Consequently, small cerebral vessels exhibit pathological hypercontraction with impaired vasodilatory capacity and reduced cerebral perfusion, culminating in ischemic-hypoxic injury to the brain tissue (Evans et al., 2021). This disrupts the activity of enzymes and function of transport proteins involved in iron metabolism. Furthermore, ferrous ions (Fe^2+^) catalyze Fenton reactions, generating reactive oxygen species. Therefore, pathological iron deposition may potentiate oxidative stress, exacerbate neuronal damage, and establish a self-perpetuating pathogenic cycle (Levi and Tiranti, 2019). In patients with VaD, chronic cerebral hypoperfusion predisposes the hippocampus to selective neuronal vulnerability, potentially driving the progressive impairment of this cognition-critical region (Zhang et al., 2023). This disruption can result in impaired iron homeostasis and clearance, facilitating iron deposition in vulnerable regions, such as the hippocampus (Long et al., 2023). The accumulation of excess iron in the hippocampus can further trigger oxidative stress in neurons, thereby accelerating degeneration and death (Gao et al., 2025). This exacerbates hippocampal atrophy and cognitive dysfunction (Yang et al., 2024).

This study has several key strengths. Firstly, we innovatively combined brain volume and iron levels to comprehensively evaluate brain atrophy and iron deposition in patients with VAD. This integrated approach is more comprehensive than single-indicator studies, and offers a new perspective on the neurobiological mechanisms of VAD. Our analysis not only revealed key brain region changes in patients with VAD, but also provided potential biomarkers for future diagnosis and treatment. Second, pathological changes in key brain regions such as the putamen, pallidum, and hippocampus in VAD were clarified. These findings are highly significant for understanding neuropathological mechanisms and identifying potential targets for VAD treatment. Third, the findings were closely linked to cognitive dysfunction in patients with VAD, such as executive and attentional deficits. These findings highlight the clinical relevance of the identified neurobiological changes and provide crucial information for future clinical intervention studies.

Nevertheless, this study has several limitations. First, the small sample size may have limited the statistical power and generalizability of the results. Future studies should therefore expand the sample size to confirm these findings and enhance their reliability. Second, the cross-sectional design made it difficult to establish causality between brain atrophy, iron deposition, and VAD. Subsequent studies should therefore adopt a longitudinal design to better track the disease progression and related brain changes. Third, although we excluded participants with any clinical features suggestive of concomitant Alzheimer's disease or other neurodegenerative disorders, we did not have access to CSF biomarkers or amyloid PET imaging. Therefore, we cannot completely exclude the possibility of mixed pathology, which is common in the elderly population. Future studies incorporating biomarker-based assessments are therefore warranted to further delineate the pathology-specific structural changes. Fourth, our imaging analyses focused specifically on the basal ganglia and hippocampus as a priori ROIs, and did not cover all brain regions (e.g., the thalamus and cortical lobes). While this focused design enhanced the statistical power and ensured consistency between volumetric and QSM analyses, it also means that structural alterations potentially present in other brain regions were not explored. Future studies using whole-brain voxel-wise analyses will be valuable to provide a more comprehensive map of the structural abnormalities in VAD. Fifth, Cognitive assessment was limited to MMSE without domain-specific tests; however, the diagnosis was established via the comprehensive DSM-5 framework, not MMSE alone. Future studies should include more detailed neuropsychological batteries. Finally, although brain volume and iron levels are common indicators of brain pathology, they may not fully capture the complex pathophysiology of VAD. Future studies should therefore incorporate multiple biomarkers, including assessments of lacunar infarcts, white matter lesions, and cerebral amyloid angiopathy, for a more comprehensive evaluation of this disease.

In conclusion, the present study shows that patients with VAD show volumes reductions in the left putamen, left pallidum, and left/right hippocampi, in addition to increased iron deposition in the left and right hippocampi. These key regional brain changes are linked to motor and cognitive issues in patients with VAD. Prior research has shown that putamen and pallidum atrophy may cause motor and cognitive deficits, while hippocampal changes may be related to memory problems. Our findings highlight the importance of these regions in VAD pathophysiology. However, this study's limitations include a small sample size and cross-sectional design. Future studies should include larger sample sizes, longitudinal designs, and multiple biomarkers.

## Data Availability

The raw data supporting the conclusions of this article will be made available by the authors, without undue reservation.
