# Supplementary material for: Subcortical gray matter atrophy and iron deposition in patients with vascular dementia: a multimodal MRI study
Source: Front Neurosci. 2026 Jul 21;20:1843628. doi: 10.3389/fnins.2026.1843628 (PMC13433758; doi:10.3389/fnins.2026.1843628)
Supplement: Supplementary file 1 [file Table_1.docx]

**Table S1.** Operational Definitions of DSM‑5 Criteria for Major Vascular Neurocognitive Disorder

| **Criterion** | **DSM‑5 Criteria** | **Operational Definition** |
| --- | --- | --- |
| A | Major neurocognitive disorder criteria met | MMSE < 24 (or < 20 if ≤6 years education), significant decline from previous level |
| B | Vascular etiology: (1) temporal relation to CVE; or (2) prominent decline in attention/processing speed and executive function | (1) Onset within 3 months post‑stroke or stepwise progression; and/or (2) impaired attention/executive function with relatively preserved memory |
| C | Cerebrovascular disease on history, exam, or imaging sufficient to explain deficits | MRI evidence of cerebrovascular disease consistent with VaD, including large-vessel infarcts, strategic infarcts (e.g., thalamus, basal ganglia), lacunar infarcts, extensive white matter hyperintensities, or hemorrhagic lesions, as assessed by neuroradiologists |
| D | Not better explained by other brain disease or systemic disorder | Exclusion of AD, DLB, FTD, hypothyroidism, B₁₂/folate deficiency, syphilis, depression |

**Table S2.** MRI scan parameters

| Sequence Parameters | T1WI | T2WI | DWI | FLATR | MPRAGE | QSM |
| --- | --- | --- | --- | --- | --- | --- |
| Sequence type | SE | TSE | RESOLVE | IR | GRE/IR | GRE |
| TR(ms) | 424 | 3600 | 4000 | 8000 | 2000 | 40 |
| TE(ms) | 6.4 | 99 | 73，120 | 117 | 2.3 | 6.84，13.12，19.40，25.68 |
| Slice thickness (mm) | 6 | 6 | 6 | 6 | 1 | 2 |
| Slice gap (mm) | 0.9 | 0.9 | 0.9 | 0.9 | 0 | 0.4 |
| Slices | 18 | 18 | 18 | 18 | 192 | 72 |
| Matrix | 320×224 | 320×224 | 160×160 | 320×272 | 256×232 | 384×255 |
| FOV(mm^2^) | 230×208 | 230×208 | 220×220 | 230×208 | 256×256 | 220×200 |

**Table S3. The domain-specific brain volume in all participants**

| **Brain regions**^*^ | **Healthy controls**  **(n=43)** | **VAD**  **(n=53)** | **P** |
| --- | --- | --- | --- |
| Left Caudate, mean (SD) | 2.25 (0.24) | 2.16 (0.45) | 0.187 |
| Left Putamen, mean (SD) | 3.26 (0.34) | 2.82 (0.50) | ＜0.001 |
| Left Pallidum, mean (SD) | 1.34 (0.13) | 1.24 (0.18) | 0.002 |
| Left Hippocampus, mean (SD) | 2.87 (0.29) | 2.60 (0.24) | ＜0.001 |
| Right Caudate, mean (SD) | 2.31 (0.26) | 2.24 (0.51) | 0.412 |
| Right Putamen, mean (SD) | 3.31 (0.33) | 2.85 (0.60) | ＜0.001 |
| Right Pallidum, mean (SD) | 1.34 (0.12) | 1.22 (0.17) | ＜0.001 |
| Right Hippocampus, mean (SD) | 3.01 (0.31) | 2.66 (0.38) | ＜0.001 |

^*^The volumes of brain regions are standardized as ratios relative to the total brain volume

**Table S4. The domain-specific brain** **iron levels in the participants with QSM**

| **Brain regions** | Healthy controls  (n=24) | VAD  (n=30) | P |
| --- | --- | --- | --- |
| Left Caudate, mean (SD) | 0.033 (0.012) | 0.031 (0.012) | 0.574 |
| Left Putamen, mean (SD) | 0.071 (0.021) | 0.082 (0.028) | 0.114 |
| Left Pallidum, mean (SD) | 0.058 (0.018) | 0.070 (0.028) | 0.065 |
| Left Hippocampus, mean (SD) | 0.002 (0.005) | 0.009 (0.005) | ＜0.001 |
| Right Caudate, mean (SD) | 0.033 (0.011) | 0.035 (0.011) | 0.584 |
| Right Putamen, mean (SD) | 0.071 (0.022) | 0.080 (0.030) | 0.200 |
| Right Pallidum, mean (SD) | 0.056 (0.018) | 0.069 (0.027) | 0.042 |
| Right Hippocampus, mean (SD) | 0.003 (0.005) | 0.009 (0.006) | ＜0.001 |
